# Supplementary material for: Recombinant Bovine Growth Hormone-Induced Metabolic Remodelling Enhances Growth of Gilthead Sea-Bream (Sparus aurata): Insights from Stable Isotopes Composition and Proteomics
Source: Int J Mol Sci. 2021 Dec 3;22(23):13107. doi: 10.3390/ijms222313107 (PMC8658469; doi:10.3390/ijms222313107)
Supplement: Supplementary file 1 [file ijms-22-13107-s001.zip › ijms-1483986-supplementary.pdf]

Title: Recombinant bovine growth hormone-induced metabolic remodelling enhances growth of gilthead sea-bream (*Sparus aurata*): insights from stable isotopes composition and proteomics

Authors:

J. Blasco, E.J. Vélez[2], M. Perelló, Sh. Azizi, E. Capilla, J. Fernández-Borràs , J. Gutiérrez

Supplementary materials

Table S1: Summary of the Gene Ontology (GO) terms of the biological processes obtained from the functional enrichment analysis for the differentially expressed proteins of white muscle after 9 weeks of rBGH injection on gilthead seabream juveniles.

Figure S1: Reproducibility of 2D-gels: CT1 to GH6.

Table S2: Raw data spots

Supplementary text: Details of LC-MS/MS analysis and Data base search

Table S1: Summary of the Gene Ontology (GO) terms of the biological processes obtained from the functional enrichment analysis for the differentially expressed proteins of white muscle after 9 weeks of rBGH injection on gilthead seabream juveniles.

Network Stats

number of nodes:	13	expected number of edges:	6
number of edges:	49	PPI enrichment p-value:	< 1.0e-16
average node degree:	7.54	*your network has significantly more interactions than expected (what does that mean?)*	
avg. local clustering coefficient:	0.846		

Functional enrichments in your network

Biological Process (GO)

GO-term	description	count in network	false discovery rate
GO:0006754	ATP biosynthetic process	8 of 75	8.19e-14
GO:0042866	pyruvate biosynthetic process	7 of 42	1.15e-13
GO:0016052	carbohydrate catabolic process	8 of 104	1.15e-13
GO:0009168	purine ribonucleoside monophosphate biosynthetic process	8 of 98	1.15e-13
GO:0006757	ATP generation from ADP	7 of 39	1.15e-13
GO:0006096	glycolytic process	7 of 38	1.15e-13
GO:0019674	NAD metabolic process	7 of 66	5.52e-13
GO:0006090	pyruvate metabolic process	7 of 66	5.52e-13
GO:0061718	glucose catabolic process to pyruvate	6 of 25	6.11e-13
GO:0061621	canonical glycolysis	6 of 25	6.11e-13
GO:0019359	nicotinamide nucleotide biosynthetic process	7 of 68	6.11e-13
GO:0006735	NADH regeneration	6 of 25	6.11e-13
GO:0046034	ATP metabolic process	8 of 190	3.02e-12
GO:0009166	nucleotide catabolic process	7 of 101	5.01e-12
GO:0017144	drug metabolic process	10 of 622	6.59e-12
GO:0006091	generation of precursor metabolites and energy	9 of 388	7.03e-12
GO:0009167	purine ribonucleoside monophosphate metabolic process	8 of 230	9.28e-12
GO:0009435	NAD biosynthetic process	6 of 51	1.30e-11
GO:0072521	purine-containing compound metabolic process	9 of 478	3.36e-11
GO:0006732	coenzyme metabolic process	8 of 297	5.31e-11
GO:0046394	carboxylic acid biosynthetic process	8 of 311	7.49e-11
GO:0090407	organophosphate biosynthetic process	9 of 577	1.53e-10
GO:0055086	nucleobase-containing small molecule metabolic process	9 of 662	4.97e-10
GO:0006006	glucose metabolic process	6 of 113	8.97e-10
GO:0005975	carbohydrate metabolic process	8 of 457	1.28e-09
GO:0006094	gluconeogenesis	5 of 46	1.42e-09
GO:0019752	carboxylic acid metabolic process	9 of 854	3.98e-09
GO:1901566	organonitrogen compound biosynthetic process	10 of 1370	7.12e-09
GO:0019637	organophosphate metabolic process	9 of 1011	1.58e-08
GO:1901135	carbohydrate derivative metabolic process	9 of 1083	2.79e-08
GO:0044281	small molecule metabolic process	10 of 1779	7.84e-08
GO:0016310	phosphorylation	9 of 1236	8.22e-08
GO:0055114	oxidation-reduction process	8 of 923	2.27e-07
GO:1901575	organic substance catabolic process	9 of 1609	7.90e-07
GO:0044248	cellular catabolic process	9 of 1646	9.43e-07
GO:1901564	organonitrogen compound metabolic process	11 of 5281	0.00020
GO:0006600	creatine metabolic process	2 of 11	0.00025
GO:0044275	cellular carbohydrate catabolic process	2 of 26	0.0012
GO:0051402	neuron apoptotic process	2 of 40	0.0026
GO:0042398	cellular modified amino acid biosynthetic process	2 of 41	0.0027

Figure S1: Reproducibility of 2D-gels: CT1 to GH6.

Focused strips were equilibrated in two steps as follows: 15 min with equilibration buffer I (65 mM DTT, 50 mM Tris-HCl, 6 M urea, 30% glycerol, 2% SDS, bromophenol blue) and then 15 min with equilibration buffer II (135 mM iodoacetamide, 50 mM Tris-HCl, 6 M urea, 30% glycerol, 2% SDS, bromophenol blue). The equilibrated strips were applied directly onto 12.5% polyacrylamide gels, sealed with 0.5% w/v agarose, and separated at a constant voltage of 50 V for 30 min followed by 200 V for about 6 h, until the blue dye reached the bottom on an Ettan DALT II system (Amersham Biosciences, Stockholm, Sweden). The resolved proteins were fixed for 1 h in 40% v/v methanol containing 10% v/v acetic acid and stained overnight using colloidal Coomassie Blue G-250.Gel staining was removed by washing steps with distilled water until the best visualization was achieved.

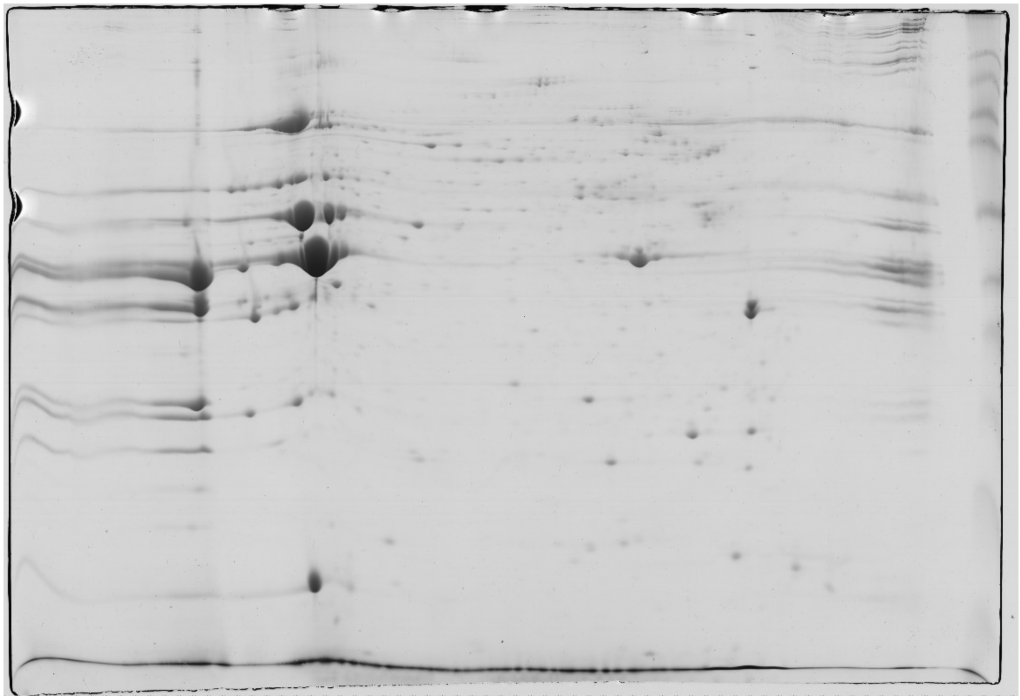

CT1

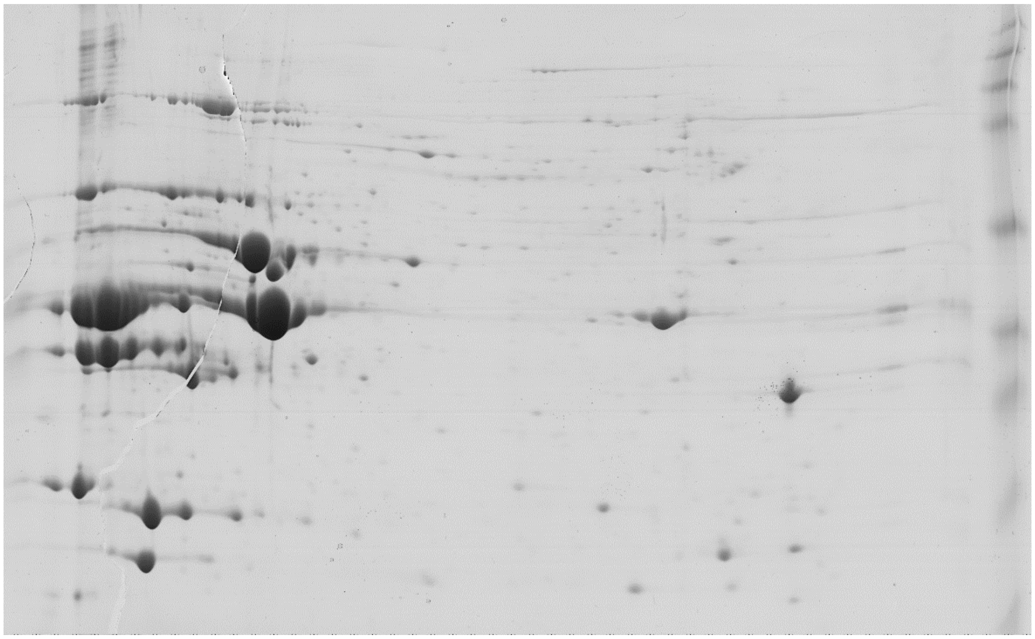

CT2

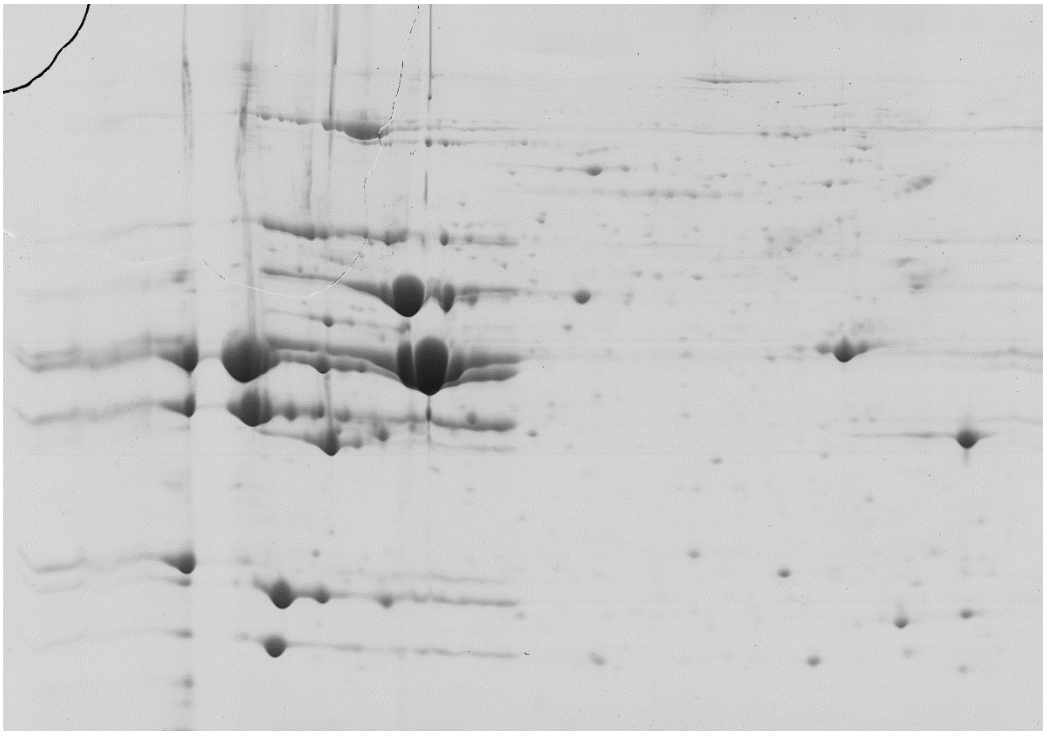

CT3

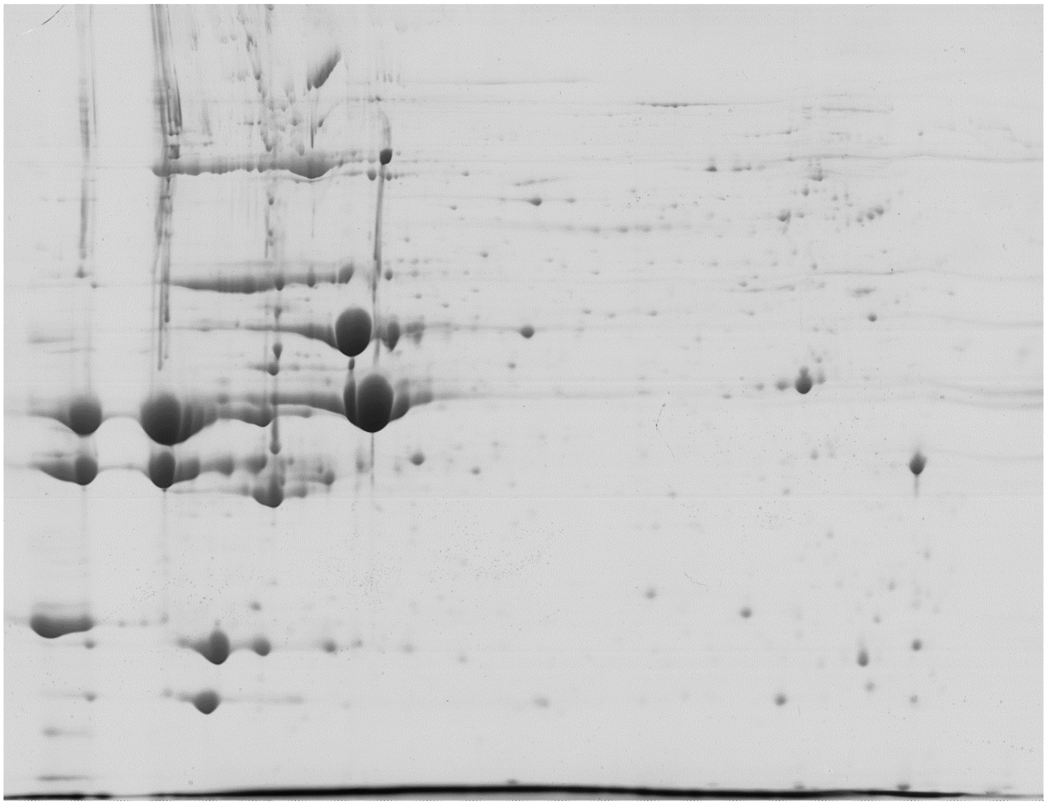

CT4

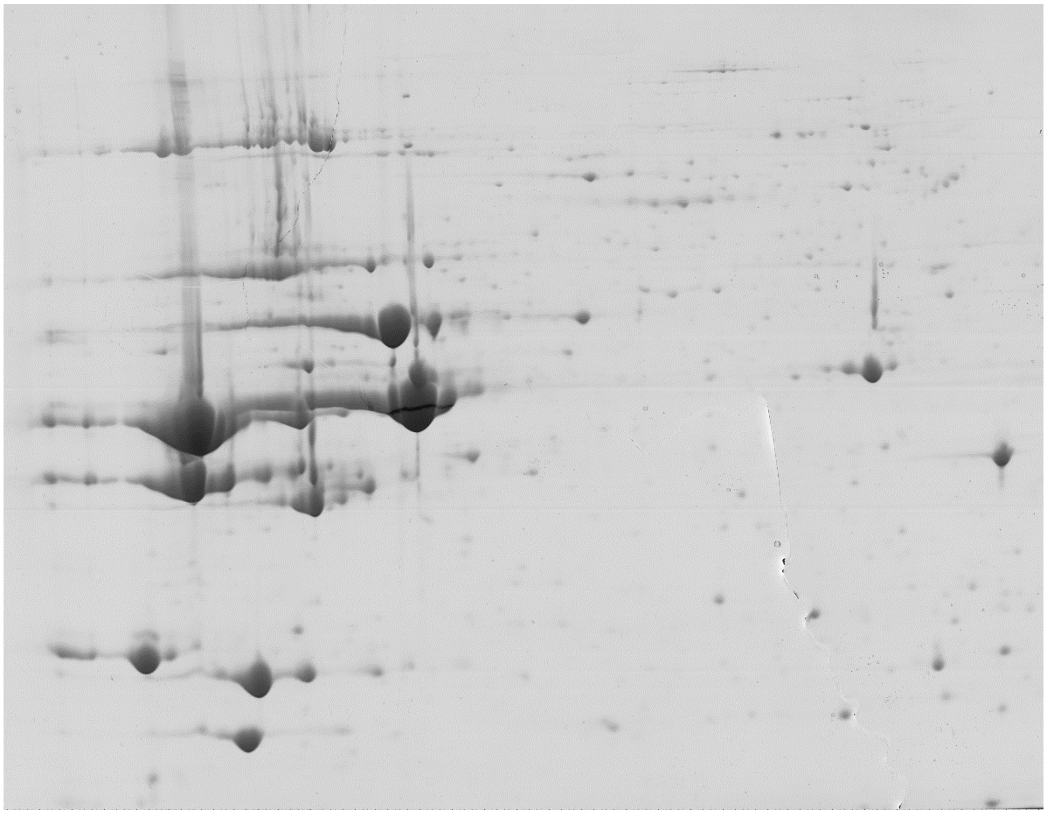

CT5

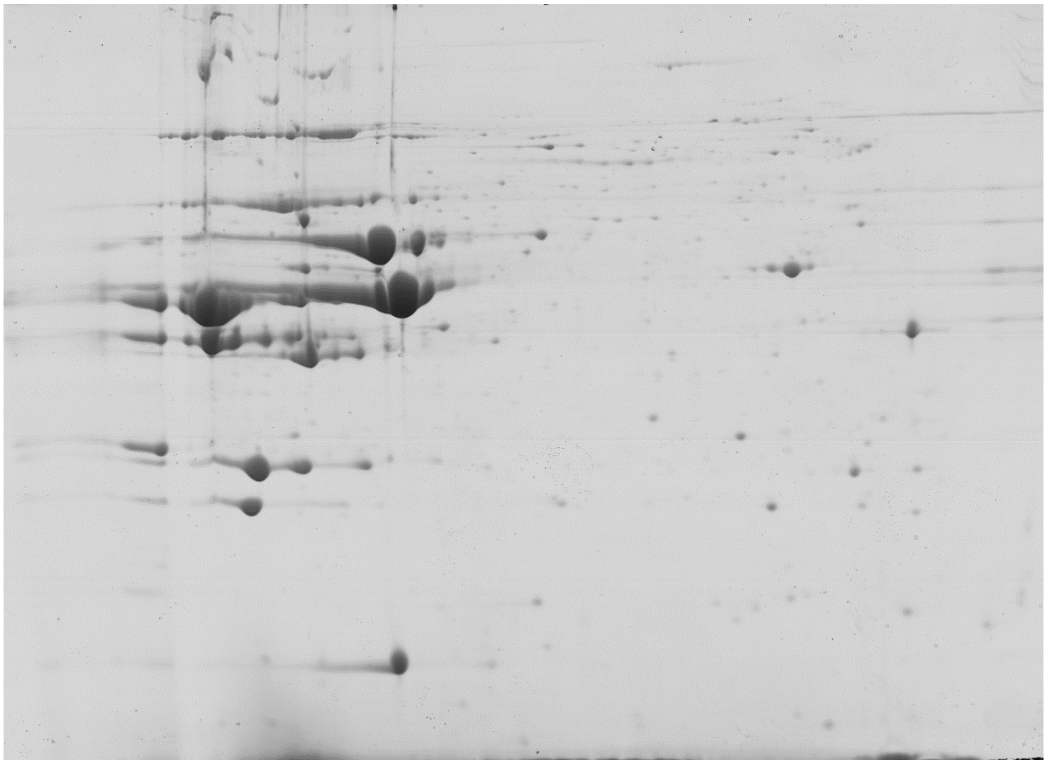

CT6

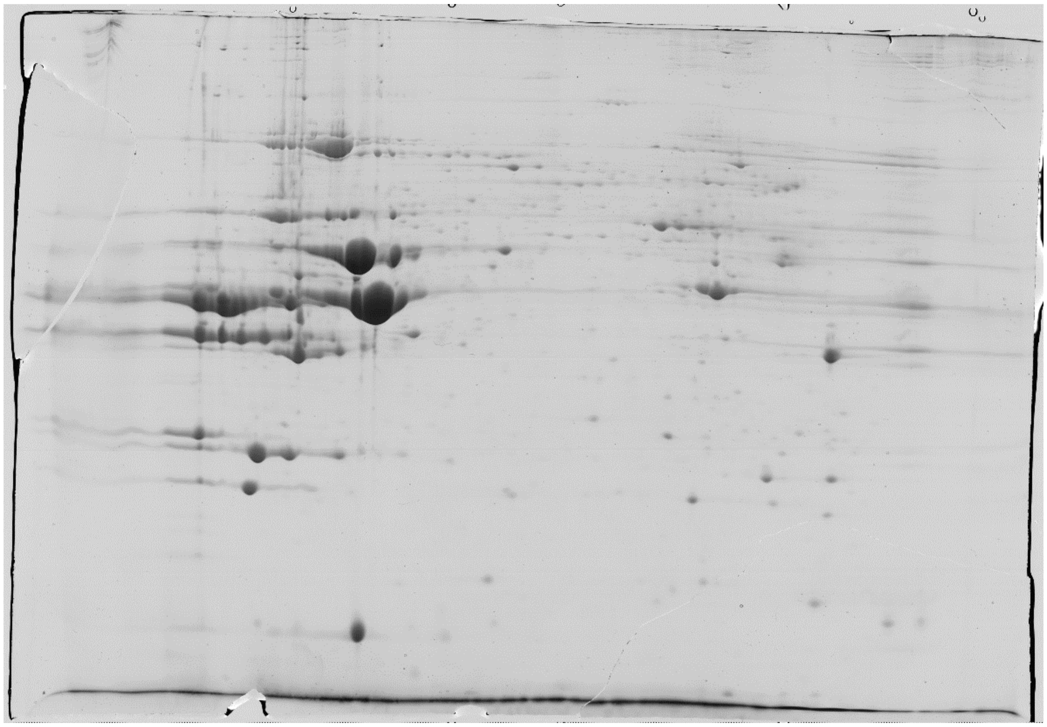

GH1

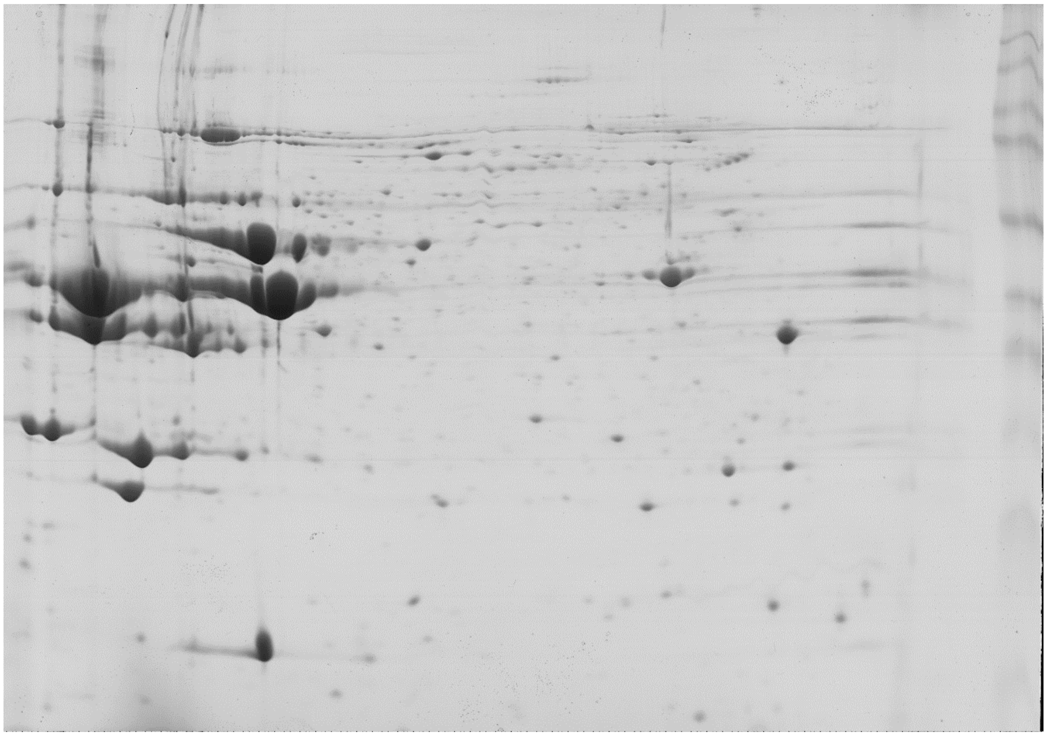

GH2

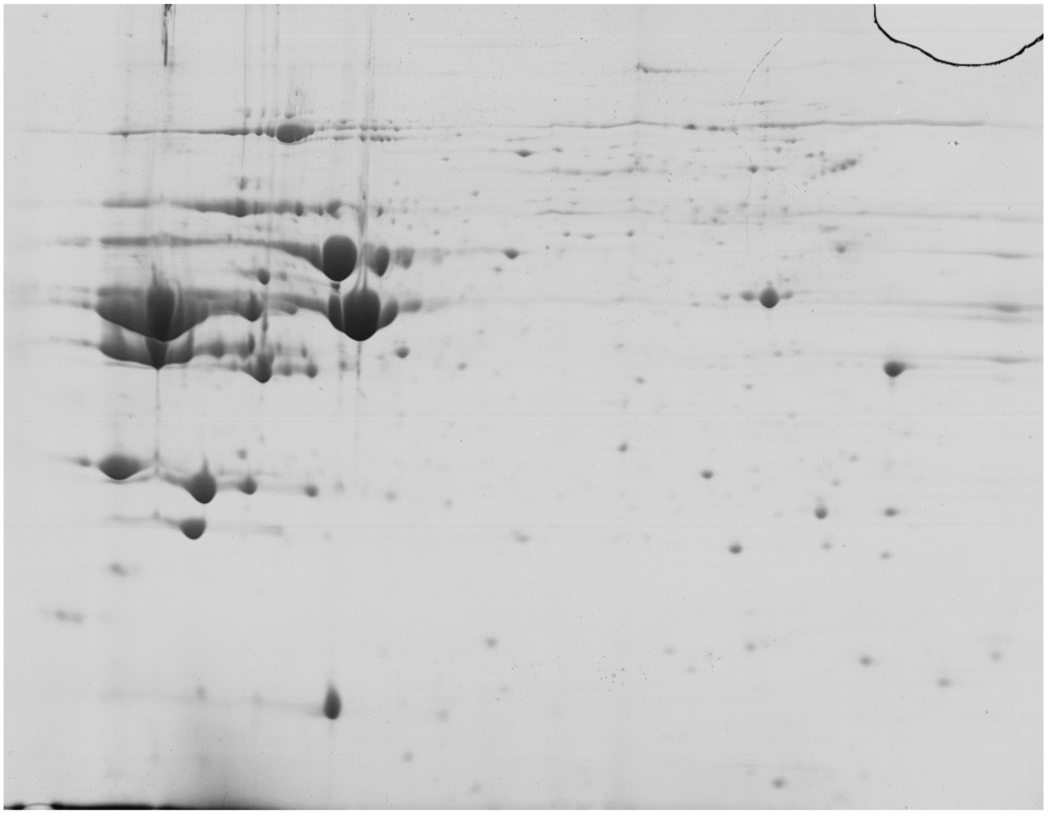

GH3

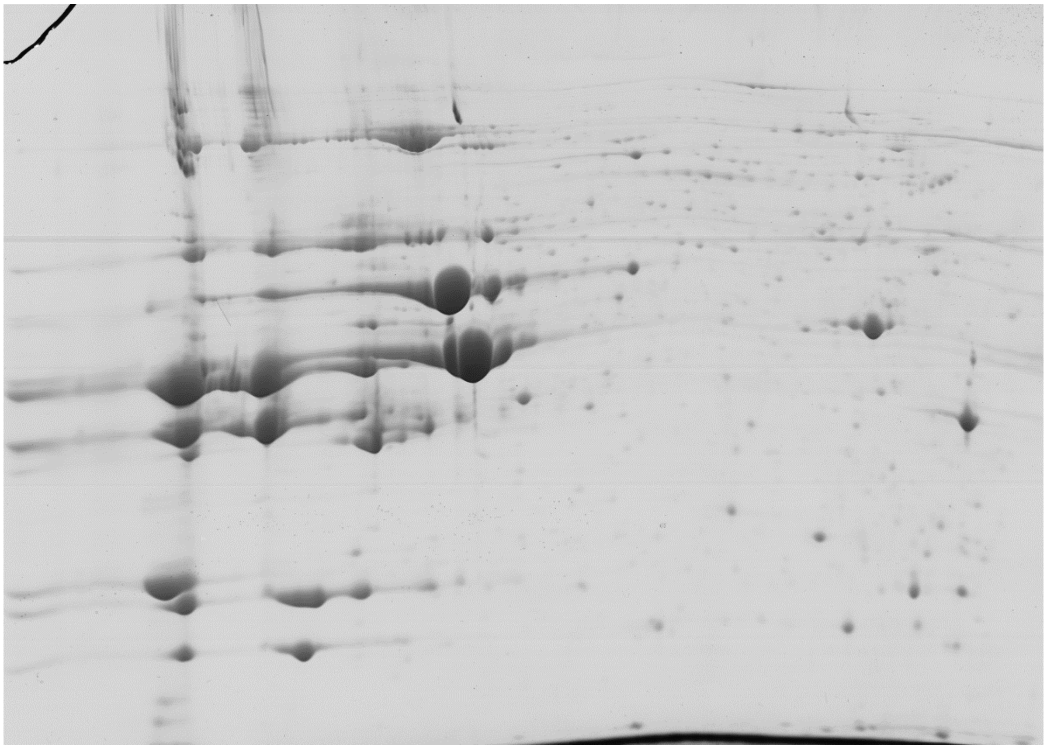

GH4

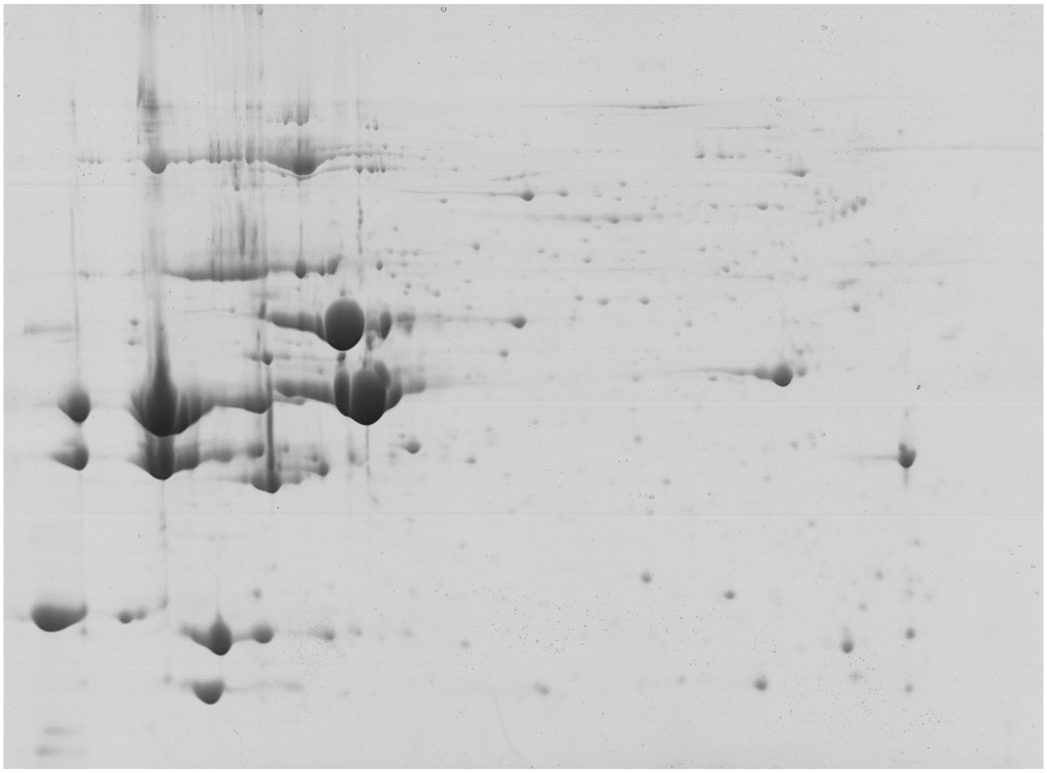

GH5

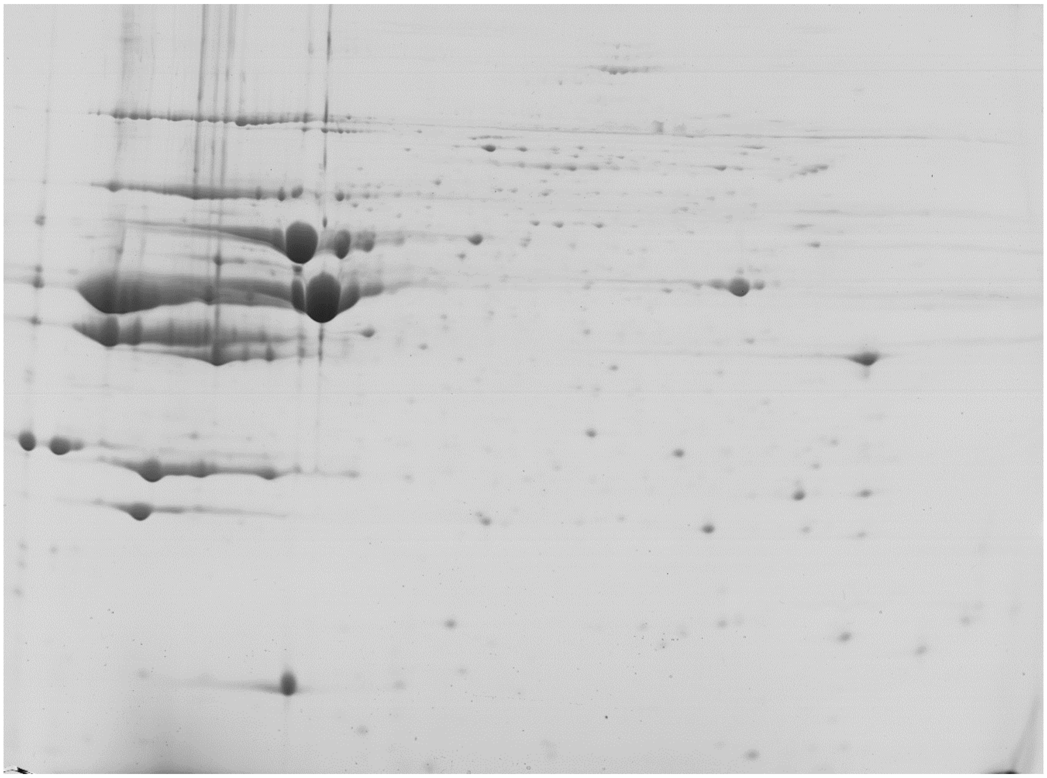

GH6

Table S2: Raw data spots

Match ID	CT1_II	CT2-II	CT3_II	CT4_II	CT5_II	MCT6_II	MeanCT	Desvest	ESM	MGH1_II	GH2_II	GH3_II	GH4_II	GH5_II	GH6_II	Mean GH	Desvest	ESM	Mean GH/Ct
494	0.526456	0.508326	0.524113	0.551016	0.600511	0.428535	0.52316	0.056471	0.023054	0.487267	0.193295	0.44393	0.514427	0.309416	0.263126	0.368577	0.131439	0.05366	0.70
157	0.190232	0.182296	0.173447	0.303621	0.157581	0.421333	0.238085	0.103862	0.042401	0.174278	0.099987	0	0.189526	0.146346	0.052625	0.11046	0.073848	0.030148	0.46
125	0.1106	0.280456	0.056559	0.176175	0.298126	0.406929	0.221474	0.130606	0.05332	0.053052	0.053052	0.053052	0.053052	0.053052	0.053052	0.053052	0	0	0.24
538	0.300832	0.25241	0.203612	0.303621	0.132027	0.406929	0.266572	0.094354	0.03852	0.320102	0.433276	0.29471	0.367448	0.393042	0.408857	0.369573	0.053308	0.021763	1.39
158	0.061936	0.154251	0.075412	0.153685	0.106474	0.396125	0.15798	0.122833	0.050147	0.078247	0.033327	0.018653	0.019339	0.079445	0.048577	0.046265	0.02751	0.011231	0.29
540	0.30968	0.392638	0.331813	0.251143	0.32368	0.392524	0.33358	0.053722	0.021932	0.246305	0.246305	0.246305	0.246305	0.246305	0.246305	0.246305	0	0	0.74
117	0.159264	0.262927	0.260171	0.269885	0.251278	0.388923	0.265408	0.073179	0.029875	0.105559	0.105559	0.105559	0.105559	0.105559	0.105559	0.105559	0	0	0.40
37	0.039816	0.217353	0.162136	0.269885	0.32368	0.381721	0.232432	0.12189	0.049761	0.081804	0.123309	0.044766	0.104433	0.112895	0.048577	0.085964	0.033387	0.01363	0.37
541	0.314104	0.343558	0.207383	0.23615	0.28109	0.345709	0.287999	0.057155	0.023334	0.436312	0.436312	0.436312	0.436312	0.436312	0.436312	0.436312	0	0	1.51
717	0.057512	0.070111	0.113118	0.161181	0.110733	0.338507	0.14186	0.103054	0.042072	0.028454	0	0	0.046415	0	0.048577	0.020574	0.023596	0.009633	0.15
909	0.13272	0.091148	0.041477	0.041233	0	0.302496	0.101512	0.108645	0.044354	0.298762	0.253283	0.436469	0.278487	0.292691	0.396713	0.326068	0.072939	0.029777	3.21
124	0.185808	0.343558	0.120659	0.074968	0.396082	0.273686	0.23246	0.126723	0.051735	0.073398	0.073398	0.073398	0.073398	0.073398	0.073398	0.073398	1.52E-17	6.21E-18	0.32
508	0.19908	0.22787	0.177218	0.333608	0.36201	0.273686	0.262245	0.074267	0.030319	0.192061	0.259948	0.141759	0.146979	0.075263	0.052625	0.144773	0.075945	0.031004	0.55
94	0.159264	0.287467	0.120659	0.048729	0.289608	0.266484	0.195369	0.100805	0.041154	0.071134	0.063325	0.14922	0.050282	0.071082	0.052625	0.076278	0.036816	0.01503	0.39
500	0.24332	0.168273	0.196071	0.153685	0.340715	0.255681	0.226291	0.068989	0.028165	0.125606	0.125606	0.125606	0.125606	0.125606	0.125606	0.125606	0	0	0.56
493	0.230048	0.238387	0.226236	0.247395	0.383305	0.25208	0.262909	0.059798	0.024413	0.192061	0	0.152951	0.228205	0	0.149779	0.120499	0.097644	0.039863	0.46
774	0.106176	0.091148	0.101806	0.026239	0.042589	0.237675	0.100939	0.074573	0.030444	0.02134	0	0	0.034811	0	0.032387	0.014756	0.016791	0.006855	0.15
681	0.482216	0.382121	0.324271	0.142439	0.340715	0.237675	0.31824	0.117394	0.047926	0.046237	0.14997	0.026114	0.116036	0.28851	0.024289	0.108526	0.101918	0.041608	0.34
728	0.08848	0.161262	0.173447	0.386086	0.353492	0.234074	0.232807	0.116218	0.047446	0.116995	0.116995	0.116995	0.116995	0.116995	0.116995	0.116995	0	0	0.50
523	0.17696	0.133216	0.214924	0.243646	0.259796	0.201664	0.205034	0.045924	0.018748	0.074691	0	0.14922	0.112168	0.150527	0.056673	0.090546	0.05849	0.023878	0.44
84	0.238896	0.04908	0.098036	0.337357	0.136286	0.194461	0.175686	0.104016	0.042464	0.05335	0.136639	0.037305	0.042547	0.058538	0.032385	0.060127	0.038735	0.015814	0.34
625	0.24332	0.196319	0.098036	0.183672	0.24276	0.19086	0.192494	0.053155	0.021701	0.081804	0.099987	0.2201	0.058018	0.108714	0.048577	0.102867	0.061949	0.025291	0.53
100	0.106176	0.133216	0.045247	0.18742	0.259796	0.183658	0.152586	0.074463	0.030399	0.05335	0	0	0.146979	0	0	0.033388	0.059599	0.024331	0.22
905	0	0.140228	0.169677	0.089962	0	0.162051	0.093653	0.077695	0.031719	0.017281	0.017281	0.017281	0.017281	0.017281	0.017281	0.017281	0	0	0.18
118	0.17696	0.171779	0.177218	0.157433	0.144804	0.147647	0.16264	0.014636	0.005975	0.039124	0	0	0.259148	0	0.036433	0.055784	0.101336	0.04137	0.34
126	0.1106	0.220859	0.026394	0.112452	0.208688	0.147647	0.137773	0.071853	0.029334	0.062163	0.062163	0.062163	0.062163	0.062163	0.062163	0.062163	0	0	0.45
169	0.057512	0.147239	0.098036	0.134943	0.132027	0.147647	0.119567	0.035385	0.014446	0.192061	0.323269	0.141759	0.154715	0.146346	0.178116	0.189378	0.068384	0.027918	1.58

279	0.123872	0.084137	0.135741	0.149936	0.255537	0.140444	0.148278	0.057333	0.023406	0.206288	0.239952	0.216369	0.193394	0.234153	0.242885	0.222174	0.020014	0.008171	1.50
764	0.039816	0.09816	0.150824	0.089962	0.140545	0.129641	0.108158	0.041067	0.016766	0.031046	0.031046	0.031046	0.031046	0.031046	0.031046	0.031046	0	0	0.29
903	0.194656	0.09816	0.196071	0.146188	0	0.129641	0.127453	0.073052	0.029823	0.049794	0.043328	0.07461	0.069622	0.029269	0.040481	0.051184	0.017591	0.007181	0.40
726	0.269864	0.189308	0.207383	0.059975	0.123509	0.12604	0.16268	0.074325	0.030343	0.065456	0.065456	0.065456	0.065456	0.065456	0.065456	0.065456	1.52E-17	6.21E-18	0.40
22	0.092904	0.09816	0.169677	0	0	0.12604	0.08113	0.068484	0.027958	0.149381	0.196627	0.29098	0.069622	0.175615	0.287414	0.19494	0.084772	0.034608	2.40
530	0.04424	0.028046	0.0641	0.213659	0.046848	0.118838	0.085955	0.069986	0.028572	0.024897	0	0	0.027075	0	0.020241	0.012035	0.013368	0.005457	0.14
734	0.075208	0.091148	0.143283	0.074968	0.102215	0.115236	0.100343	0.026189	0.010692	0.065091	0.065091	0.065091	0.065091	0.065091	0.065091	0.065091	0	0	0.65
206	0.101752	0.101665	0.041477	0.097459	0.093697	0.108034	0.090681	0.024578	0.010034	0.058132	0.058132	0.058132	0.058132	0.058132	0.058132	0.058132	0	0	0.64
727	0.04424	0.056091	0.12443	0.269885	0.310903	0.108034	0.152264	0.111938	0.045698	0.366339	0.293294	0.36559	0.212733	0.309416	0.202405	0.29163	0.071493	0.029187	1.92
875	0.234472	0.112182	0.211153	0.127446	0	0.104433	0.131614	0.084098	0.034333	0.039124	0	0	0.05415	0	0.08501	0.029714	0.035755	0.014597	0.23
36	0.115024	0.136722	0.25263	0.198666	0.153322	0.104433	0.160133	0.056165	0.022929	0.08542	0.08542	0.08542	0.08542	0.08542	0.08542	0.08542	0	0	0.53

Supplementary text

Details of LC-MS/MS analysis

The dried-down peptide mixtures were analysed in a nanoAcquity liquid chromatographer (Waters, Mildorf, MA, USA)) coupled to a LTQ-Orbitrap Velos (Thermo Scientific, Bremen, Germany) mass spectrometer. The tryptic digests were resuspended in 1% FA solution and an aliquot was injected for chromatographic separation. Peptides were trapped on a Symmetry C18TM trap column (5µm 180µm x 20mm; Waters), and were separated using a C18 reverse phase capillary column (ACQUITY UPLC M-Class Peptide BEH column; 130A, 1.7µm, 75 µm x250 mm, Waters). The gradient used for the elution of the peptides was 1 to 40 % B in 25 minutes, followed by gradient from 40% to 60% in 5min (A: 0.1% FA; B: 100% ACN, 0.1%FA), with a 250nL/min flow rate. Eluted peptides were subjected to electrospray ionization in an emitter needle (PicoTipTM, New Objective) with an applied voltage of 2000V. Peptide masses (m/z 300-1700) were analyzed in data dependent mode where a full Scan MS was acquired in the Orbitrap with a resolution of 60,000 FWHM at 400m/z. Up to the 10th most abundant peptides (minimum intensity of 500 counts) were selected from each MS scan and then fragmented in the linear ion trap using CID (38% normalized collision energy) with helium as the collision gas. The scan time settings were: Full MS: 250 ms (1 microscan) and MSn: 120 ms. Generated .raw data files were collected with Thermo Xcalibur (v.2.2).

Details of Data base search

The .raw files obtained in the mass spectrometry analyses were used to search against the public database Uniprot Actinopterygii (v.23/3/17). A database containing common laboratory contaminant proteins was added to this database. The software used was Thermo Proteome Discoverer (v.1.4.1.14) with Sequest HT as the search engine. The following search parameters were applied: Database/Taxonomy: Uniprot_Actinopterygii_cont; Enzyme specificity: Trypsin Max. miscleavage sites 2; Fixed modifications: Carbamidomethyl (C); Variable modifications: Oxidation (M); Peptide tolerance: 10 ppm and 0.6Da (respectively for MS and MS/MS spectra); Percolator Target FDR (Strict): 0.01; Validation based on: q-Value. Both a target and a decoy database were searched in order to obtain a false discovery rate (FDR), and thus estimate the number of incorrect peptide-spectrum matches that exceed a given threshold. The search results were visualized in Proteome Discoverer (v.1.4.1.14) and exported to Excel as lists of identified proteins. The results have been filtered so only proteins identified with at least 2 high confidence (FDR> 1%) peptides are included in the lists. All possible protein identifications from analyses that met the above criteria were reported for each gel spot. However, the protein identification with the highest score, discarding contaminants, was selected in the case of redundant protein identifications. The Batch-Genes tool produced by GOEAST (http://omicslab.genetics.ac.cn/GOEAST/) performed enrichment analyses of Gene Ontology (GO) annotation terms for biological processes. The proteomics work was done at the Proteomics Platform of Barcelona Science Park, University of Barcelona, a member of ProteoRed-ISCIII network.
